# Inducing disulfidptosis in tumors:potential pathways and significance

**DOI:** 10.1002/mco2.791

**Published:** 2024-10-15

**Authors:** Tao Mi, Xiangpan Kong, Meiling Chen, Peng Guo, Dawei He

**Affiliations:** ^1^ Department of Urology Children's Hospital of Chongqing Medical University National Clinical Research Center for Child Health and Disorders Ministry of Education Key Laboratory of Child Development and Disorders Chongqing P.R. China; ^2^ Chongqing Key Laboratory of Structural Birth Defect and Reconstruction Chongqing P.R. China; ^3^ Institute of Basic Medicine and Cancer (IBMC) Chinese Academy of Sciences Hangzhou P.R. China

**Keywords:** cystine, disulfidptosis, NADPH, SLC7A11, tumor immunity

## Abstract

Regulated cell death (RCD) is crucial for the elimination of abnormal cells. In recent years, strategies aimed at inducing RCD, particularly apoptosis, have become increasingly important in cancer therapy. However, the ability of tumor cells to evade apoptosis has led to treatment resistance and relapse, prompting extensive research into alternative death processes in cancer cells. A recent study identified a novel form of RCD known as disulfidptosis, which is linked to disulfide stress. Cancer cells import cystine from the extracellular environment via solute carrier family 7 member 11 (SLC7A11) and convert it to cysteine using nicotinamide adenine dinucleotide phosphate (NADPH). When NADPH is deficient or its utilization is impaired, cystine accumulates, leading to the formation of disulfide bonds in the actin cytoskeleton, triggering disulfidptosis. Disulfidptosis reveals a metabolic vulnerability in tumors, offering new insights into cancer therapy strategies. This review provides a detailed overview of the mechanisms underlying disulfidptosis, the current research progress, and limitations. It also highlights innovative strategies for inducing disulfidptosis and explores the potential of combining these approaches with traditional cancer therapies, particularly immunotherapy, to expedite clinical translation.

## Graphical abstract

Exploring the mechanisms of disulfidptosis reveals potential pathways to induce tumor cell death. Tumor cells with high SLC7A11 expression uptake a large amount of cystine to meet their metabolic needs. The reduction of cystine requires the involvement of the Trx system and NADPH. When NADPH is deficient or the Trx system is dysfunctional, cystine and other disulfides induce actin cytoskeleton disulfide stress, leading to disulfidptosis. Rac‐WRC‐Arp2/3 mediates actin polymerization, facilitating disulfidptosis. In this process, targeting NADPH metabolism‐related enzymes and the Trx system is the most promising method to induce disulfidptosis.

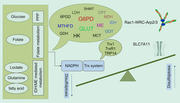

## 1 INTRODUCTION

One of the major challenges in the fight against cancer is that cancer cells can evade the normal rules of cell death and proliferate rapidly. RCD refers to a controlled and orderly cell death process under genetic regulation, aimed at maintaining homeostasis. It plays a crucial role in inducing cancer cell death, thereby contributing to cancer treatment.^1^ Apoptosis was the first identified form of RCD, and therapies that induce apoptosis in cancer cells have achieved success. However, cancer cells often develop resistance to such treatments, leading to treatment resistance and relapse.^2^ As a result, scientists are seeking more effective anticancer strategies based on other cell death mechanisms, such as ferroptosis, autophagy, and pyroptosis. Similarly, tumors have evolved different resistance strategies to these forms of RCD.^1^ Disulfidptosis, a newly discovered form of cell death, may offer new opportunities for cancer therapy.^3^

Unlike traditional apoptosis, necrosis, or autophagy, disulfidptosis is characterized by the collapse of the cytoskeleton due to intracellular disulfide stress.^3^, ^4^ This concept emerged from extensive studies on the function of the solute carrier family 7 member 11 (SLC7A11) in cancer cells.^4^, ^5^, ^6^ SLC7A11 (also known as xCT), an antiporter, imports cystine into the cytoplasm while exporting glutamate, playing a crucial role in tumors in countering oxidative stress.^4^, ^7^ Compared with nontumor cells, cancer cells frequently endure higher levels of oxidative stress, which requires cancer cells maintain sufficient levels of Glutathione (GSH) to neutralize the excess reactive oxygen species (ROS) within.^8^, ^9^, ^10^ In the synthesis of GSH, cysteine, which is instable and exists outside the cell in the form of cystine, acts as a key substrate.^11^ Thus, most tumor cells rely predominantly on SLC7A11 to transport extracellular cystine into the cytoplasm and use nicotinamide adenine dinucleotide phosphate (NADPH) to reduce it back to cysteine.^12^ In recent years, SLC7A11 has gained recognition for its crucial role in preventing ferroptosis by enhancing intracellular GSH levels, thereby positioning it as a cancer‐promoting oncogene.^9^, ^13^, ^14^ However, in 2017, a surprising aspect of SLC7A11 was unveiled when two independent research groups reported its role in promoting cell death under conditions of glucose deprivation.^15^, ^16^ Subsequent studies also highlighted the synthetic lethal effect of high SLC7A11 expression combined with glucose starvation.^17^ By 2023, Liu et al.^3^ formally defined this mode of death as disulfidptosis and unveiled its underlying mechanisms.

Since its discovery, disulfidptosis has generated significant interest in the scientific community, given its potential role in disease diagnosis, prognostic stratification, and precision therapy. Within just two years, more than 200 related studies have emerged. As research into the mechanisms of disulfidptosis deepens, growing evidence suggests its involvement in tumor progression,^3^, ^18^ neurological disorders,^19^, ^20^ and bone metabolism abnormalities.^21^ Therefore, a thorough understanding of disulfidptosis holds substantial clinical significance for the understanding and treatment of these diseases. However, despite significant discoveries, research on disulfidptosis is still in its early stages. Many questions remain unresolved, and inducing disulfidptosis in a way that benefits patients clinically requires further theoretical and experimental exploration.

This review provides a detailed examination of the mechanisms underlying disulfidptosis, discusses current progress and limitations, and identifies cystine accumulation as a key factor in the initiation of disulfidptosis. By focusing on this crucial aspect, we aim to explore methods for inducing disulfidptosis and accelerate its translation into clinical practice.

## 2 MOLECULAR MECHANISMS OF DISULFIDPTOSIS

### 2.1 Advances in the study of disulfidptosis

Disulfidptosis is found related to the balance of cystine and NADPH. Initially, Liu et al.^3^ observed rapid cell death in SLC7A11‐overexpressing cells under glucose starvation conditions. Given that glucose is the primary energy source for cells, they investigated whether ATP deficiency was the cause of cell death. Subsequent studies have shown that the deficiency of NADPH, rather than ATP, leads to this type of cell death.^3^ SLC7A11‐overexpressing cells uptake excessive cystine, which provides abundant precursors for the synthesis of the antioxidant GSH, helping cells resist oxidative stress. However, this comes at a cost, as cystine reduction back to cysteine requires NADPH.^22^, ^23^ In scenarios like glucose starvation, where NADPH is insufficient, cystine accumulates, leading to cell death.^3^ Researchers speculated that all factors inducing NADPH deficiency might lead to this type of cell death, although this has not been fully confirmed.

Liu et al.^3^ further investigated how the accumulation of cystine due to NADPH deficiency leads to cell death. They discovered that treatments preventing disulfide accumulation could inhibit the death of cells with high SLC7A11 expression under glucose deprivation conditions, thereby linking this type of cell death to disulfide stress. The authors then employed a bioorthogonal chemical proteomics strategy to quantitatively analyze changes in the disulfide proteome in glucose‐deprived, SLC7A11‐overexpressing UMRC6 cells. Gene Ontology (GO) analysis revealed significant enrichment of biological processes or pathways related to the actin cytoskeleton and cell adhesion among the disulfide‐bonded proteins induced by glucose starvation.^3^

To further elucidate the mechanisms of disulfide stress‐induced cell death, A genome‐wide CRISPR screen was conducted on cancer cells overexpressing SLC7A11 under both glucose‐sufficient and glucose‐deficient conditions. NCK‐associated protein (NCKAP1), alongside SLC7A11 and SLC3A2, was identified as one of the top suppressors, with its loss alleviating disulfidptosis. NCKAP1 is a critical component of the WAVE regulatory complex (WRC), which activates the actin‐related protein 2 and 3 (Arp2/3) complex, leading to branched actin polymerization and lamellipodia formation, regulated by Rac signaling. Further functional studies demonstrated that the loss of NCKAP1 and other WRC proteins mitigates disulfidptosis, whereas the overexpression of constitutively active Rac promotes disulfidptosis in a WRC‐dependent manner.^3^ This may be due to the branched actin network in lamellipodia, generated by the Rac‐WRC pathway, providing essential targets for disulfide bond formation between actin cytoskeleton proteins.^3^, ^24^

In conclusion, Liu et al.^3^ have identified a novel mode of cell death that is facilitated by cytoskeletal disulfide bond formation due to cystine accumulation.

### 2.2 Limitations in the study of disulfidptosis

Despite the significant achievements made by researchers in exploring the novel phenomenon of disulfidptosis, several questions remain unresolved in this process. For instance, whether all methods of inducing NADPH depletion can lead to disulfidptosis is still unclear. Besides, several aspects of disulfide bonding also require further exploration.

First, while Liu et al.^3^ identified NADPH deficiency as the primary factor preventing cystine from converting back to cysteine, leading to cystine accumulation, the exact mechanism of this conversion was not elucidated in the initial studies. A subsequent study revealed that Thioredoxin‐related protein of 14 kDa (TRP14) is a key enzyme in converting cystine to cysteine, and TRP14 requires TrxR to maintain its reduced state using NADPH as a substrate.^22^ Therefore, even with sufficient NADPH, inhibiting TrxR can still induce disulfidptosis.^21^, ^23^ Other studies have indicated that Trx1 also plays varying roles in reducing cystine, which has been overlooked and requires further investigation.^22^, ^25^

Second, GO analysis identified significant enrichment in biological processes related to the actin cytoskeleton and cell adhesion, yet other pathways such as “Enzyme binding” and “RNA binding” were also significantly enriched, but their roles were not explained.^3^ In another study on disulfide stress, researchers found that ribonuclease inhibitors and serine/threonine phosphatase PP2A were oxidized in acute pancreatitis. This suggests that the cytoskeleton is not the sole target of disulfide stress.^26^ There is no direct evidence that disulfide bond formation in the cytoskeleton is the cause of cell death. The inhibition of cell death by eliminating disulfide bonds also eliminates disulfide bonds in other functional proteins, making it difficult to assess their roles. In addition, Liu et al.^3^ highlighted the importance of WRC but neglected the roles of other genes such as RPN1, which was ranked higher than NCKAP1 as a suppressor hit.

Third, the specific processes involved in the formation and inhibition of disulfide stress are not fully understood. Whether cytosolic protein disulfide bonds form spontaneously or require enzymatic assistance, and how they are reduced by NADPH, require further research. The thioredoxin system not only plays a role in reducing cystine but may also be crucial in reducing protein disulfide bonds, necessitating further elucidation of its functions.^27^

In any case, Liu et al.^3^ have established a significant research area, and despite the current challenges, this direction remains crucial for guiding future research.

### 2.3 Key regulatory pathways of disulfidptosis

Several pathways may be closely associated with disulfidptosis. As previously noted, the Rac‐WRC pathway may play a crucial role in the final stages of this cell death mechanism.^3^ However, further studies are needed to elucidate its precise assembly in vivo, its regulation of disulfidptosis, and its potential as a therapeutic target for specific diseases.

Since SLC7A11 is a major determinant of cellular sensitivity to disulfidptosis, pathways that influence SLC7A11 expression are closely linked to this mechanism. Two key regulators of SLC7A11 transcription are activating transcription factor 4 (ATF4) and nuclear factor erythroid 2‐related factor 2 (Nrf2).^28^ ATF4 binds to the amino acid response element (AARE), promoting the transcription of stress‐response genes, including SLC7A11.^29^ Under basal conditions, Nrf2 is ubiquitinated by the E3 ligase Keap1, leading to its instability. Oxidative stress disrupts Keap1‐mediated degradation, allowing Nrf2 to bind to the antioxidant response element, thereby supporting antioxidant defenses and redox homeostasis.^30^ Additionally, the p53 signaling pathway directly inhibits SLC7A11 transcription, suppressing ferroptosis.^13^

The AMPK pathway may be among the pathways most closely related to disulfidptosis. AMPK regulates NADPH homeostasis and promotes tumor cell survival under energy‐stress conditions, such as glucose deprivation.^31^ Notably, lung cancer cells with LKB1 mutations are particularly susceptible to glucose starvation‐induced cell death, with over 50% of H460 cells (a lung cancer cell line with LKB1 inactivation) dying within six hours of glucose deprivation.^32^ However, this form of cell death is not a conventional programmed cell death; it is hypothesized to be disulfidptosis. This suggests that targeting both glucose metabolism and the AMPK signaling pathway may synergistically induce disulfidptosis, leading to enhanced antitumor effects.

Furthermore, as both NADPH reduction and inhibition of the Trx system can promote disulfidptosis, regulatory pathways involving these factors, such as the PI3K‐AKT pathway^33^ and the Toll‐like receptor pathway,^34^ may influence cell death. However, these pathways generally exert indirect or nonspecific effects, and further evidence is required to establish their relationship with disulfidptosis.

In summary, several pathways are closely associated with disulfidptosis sensitivity, and inhibitors of these pathways may induce disulfidptosis either independently or in combination with other therapies, presenting potential for enhanced antitumor strategies.

### 2.4 The relationship between disulfide stress, oxidative stress, and disulfidptosis

Despite some unresolved questions surrounding disulfidptosis, it holds significant therapeutic potential for diseases, much like other forms of RCD. A deeper understanding of disulfidptosis requires distinguishing it from other similar concepts, such as disulfide stress and oxidative stress.

Disulfide stress refers to the excessive formation of protein disulfide bonds in the cytoplasm, leading to a series of protein dysfunctions. Severe disulfide stress can result in cell death through disulfidptosis.^3^, ^24^ Oxidative stress is defined as the excessive production of ROS.^35^, ^36^ Disulfide stress can be triggered by oxidative stress. In 2014, Juan Sastre's team was the first to report cystine‐mediated disulfide stress in mammalian cells, classifying it as a specific type of oxidative stress.^26^ This classification is somewhat justified, though it may not be entirely accurate. ROS can induce various types of cell death depending on the conditions, such as apoptosis, ferroptosis, and autophagy.^37^, ^38^ Subsequent studies have shown that moderate overexpression of SLC7A11 benefits cancer cells treated with H2O2, a common oxidative stress inducer, by inhibiting oxidative stress‐induced apoptosis. However, high overexpression of SLC7A11 significantly increases H2O2‐induced cell death, leading to disulfidptosis.^4^

Therefore, oxidative stress can indeed trigger disulfide stress then leading to disulfidptosis, primarily by depleting NADPH and causing cystine accumulation. However, oxidative stress is one of several factors that can induce disulfide stress, but it is not the sole contributor.

### 2.5 Endoplasmic reticulum stress and disulfidptosis

Since both involve disulfide bond formation, endoplasmic reticulum (ER) stress can easily be confused with disulfidptosis. Under physiological conditions, disulfide bonds are formed during the folding of nascent polypeptides into their native protein structures—a process known as oxidative protein folding, which primarily occurs in the lumen of the ER in eukaryotic cells.^39^ This process is critical for maintaining the functional and structural stability of proteins, particularly secretory proteins.^40^ Proper disulfide bond formation requires enzymes to introduce disulfides between proximal cysteine residues and to reduce any disulfides formed during folding that are not present in the final native structure. Thus, during protein folding, both reductive and oxidative pathways must coexist in the ER to ensure the correct formation of native disulfides and the elimination of nonnative ones. The protein disulfide isomerase (PDI) family is the key pathway in the ER of eukaryotic cells that catalyzes oxidative protein folding.^41^, ^42^ To drive disulfide formation, PDI family members themselves must be oxidized. It is now well established that several pathways can oxidize disulfide exchange proteins via specific oxidases, with ERO1 being the primary enzyme involved. Other enzymes include glutathione peroxidases 7 and 8 and peroxiredoxin IV.^42^ PDI family members can also catalyze the reduction of disulfides; however, to do so, a pathway to reduce PDI is required. Although it remains unclear whether a specific enzymatic system exists to reduce PDI in the ER, the glutathione redox buffer seems to play a role in this process.^41^

Under various physiological and pathological conditions, such as glucose deprivation, the ER's capacity to manage protein folding can be easily overwhelmed. When proteins fail to achieve their native conformations, leading to an accumulation of unfolded proteins within the ER lumen, the condition known as ER stress arises, disrupting cellular homeostasis. To counteract this, cells initiate the unfolded protein response (UPR), a series of adaptive mechanisms aimed at restoring homeostasis.^43^ However, if ER stress is prolonged and severe, the UPR may trigger cell death pathways, including apoptosis or autophagy.^44^

Disulfide bond formation under disulfide stress primarily occurs in the cytoplasm and may arise stochastically under oxidative conditions. Research has demonstrated that oxidized glutathione (GSSG) and hydrogen peroxide (H₂O₂) can directly induce disulfide bond formation in unfolded proteins in vitro.^45^, ^46^, ^47^ The reduction of cytoplasmic disulfide bonds is likely mediated predominantly by the Trx system,^48^, ^49^ which will be elaborated on in subsequent sections.

In summary, while both disulfide stress and ER stress result from the formation of erroneous disulfide bonds, the sites of occurrence and their underlying mechanisms differ significantly.

### 2.6 Advantage in inducing disulfidptosis in tumors

The mechanism of disulfidptosis reveals its metabolic vulnerabilities. Although some aspects remain unclear, it is evident that disulfidptosis holds significant potential for cancer therapy. Inducing tumor disulfidptosis offers two major advantages. First, its metabolic dependency allows for selective targeting of cancer cells while sparing normal cells, thereby reducing drug side effects. Second, tumors resistant to conventional treatments may be more susceptible to disulfidptosis. For example, cancer cells exhibit higher glucose uptake and resist apoptosis and ferroptosis by overexpressing SLC7A11, leading to chemotherapy resistance.^28^ Depriving glucose in SLC7A11‐high tumors can rapidly induce disulfidptosis, to which normal cells are more tolerant.^3^ Additionally, the crucial role of SLC7A11 enables rapid identification of tumor subtypes sensitive to disulfidptosis, facilitating precision therapy.

In conclusion, inducing disulfidptosis represents a promising new approach to cancer treatment. The current challenge lies in effectively inducing disulfidptosis, which will be thoroughly discussed in the following sections.

## 3 POTENTIAL METHODS TO INDUCE DISULFIDPTOSIS IN TUMORS

**FIGURE 1:**
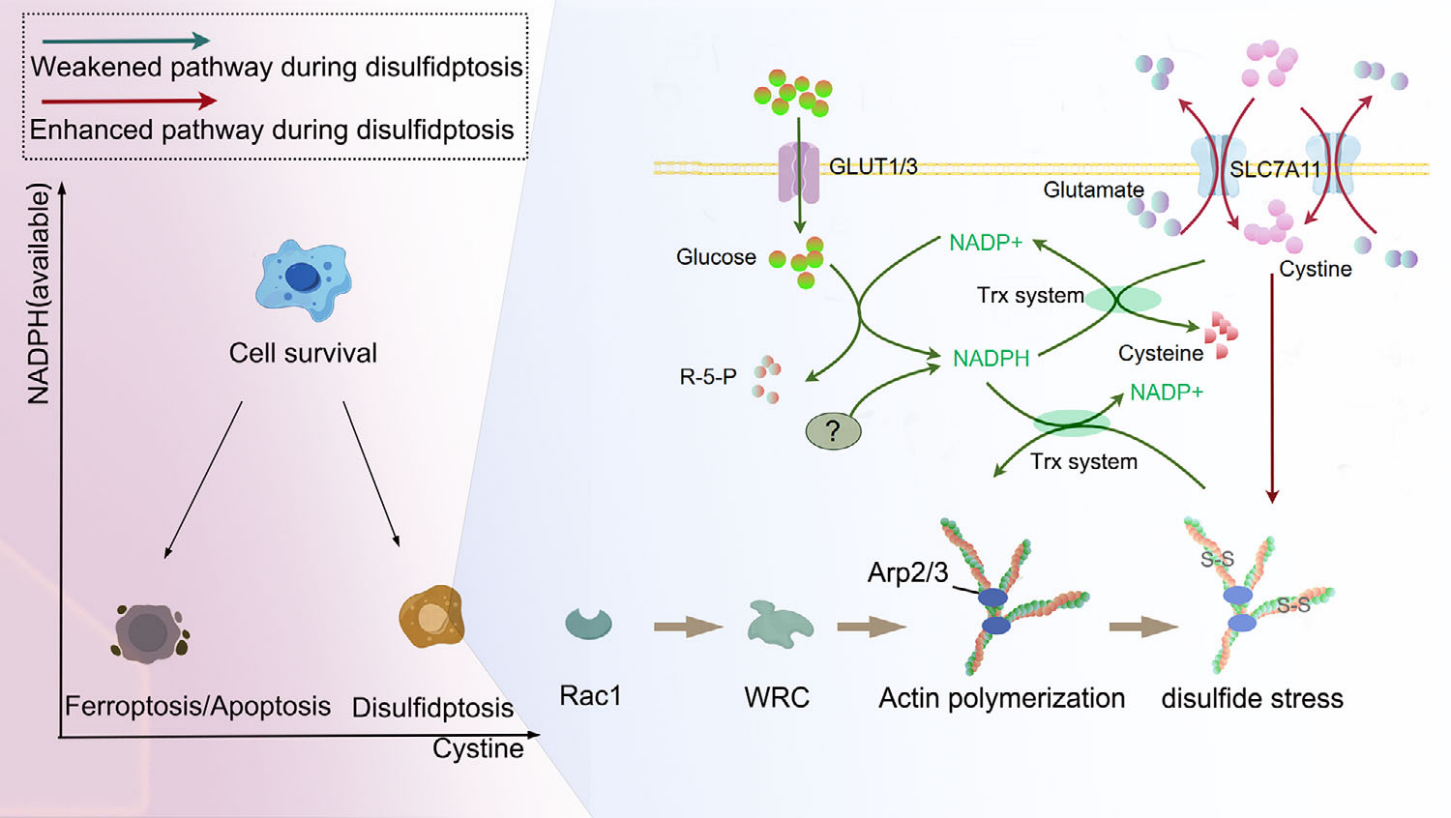
Mechanism of disulfidptosis. Cells with high expression of SLC7A11 take up excessive cystine to combat oxidative stress. Once inside the cell, cystine requires the thioredoxin system, using NADPH as a substrate, to be reduced to cysteine. When NADPH is depleted or the thioredoxin system is impaired, cystine cannot be reduced, leading to its accumulation. The accumulated cystine, as a disulfide compound, triggers the formation of disulfide bonds within the actin cytoskeleton proteins, causing its collapse and ultimately resulting in disulfidptosis. The formation of f‐actin networks is associated with Rac‐WRC‐Arp2/3 mediated actin accumulation. Importantly, both excessive and insufficient cystine levels can lead to cell death under available NADPH shortage, where insufficient cystine, coupled with reduced available NADPH, can induce apoptosis or ferroptosis due to exacerbated oxidative stress. GLUT, glucose transporters; PPP, pentose phosphate pathway; R5P, ribose‐5‐phosphate; SLC7A11, solute carrier family 7 member 11; WRC, WAVE regulatory complex; Arp2/3, Actin‐related protein 2/3; NADPH, nicotinamide adenine dinucleotide phosphate; Rac1, Ras‐related C3 botulinum toxin substrate 1.

The induction of disulfidptosis in tumor cells through glucose transporter (GLUT) inhibitors has shown promising potential for clinical applications.^4^ This success also prompts us to consider whether other methods could similarly induce disulfidptosis. Before delving into this question, we first review and summarize the mechanisms. Three conditions may be required to induce disulfidptosis^3^, ^50^: (1) Excessive cystine intake, where the key factor is cystine overload rather than merely high SLC7A11 expression, as extra cystine can also trigger disulfidptosis in tumor cells with low SLC7A11; (2) deficiency or impaired utilization of NADPH, preventing adequate conversion of cystine to cysteine; (3) accumulated cystine causing abnormal disulfide bond formation in the actin cytoskeleton (Figure 1).

These three criteria define the conditions necessary to induce disulfidptosis, providing new insights for cancer therapy. Currently, disulfidptosis is chiefly triggered by the second criterion—glucose starvation or inhibition of glucose transporters, which restricts NADPH production in tumor cells with high SLC7A11 expression.^3^ Glucose deprivation initiates disulfidptosis as it reduces the substrate available for the pentose phosphate pathway (PPP), the main route for NADPH generation in most cells.^51^ Moreover, disulfidptosis can be induced by depleting NADPH via H_2_O_2_.^4^ It remains uncertain whether alternative methods of reducing NADPH, such as inhibiting isocitrate dehydrogenase (IDH) and malic enzyme (ME), can also induce disulfidptosis.^52^, ^53^ Moreover, in the process of inhibiting cystine conversion, the Trx system—often overlooked but critical for cystine reduction—deserves greater attention. Inhibiting these enzymes has been shown to induce disulfidptosis.^21^ In fact, blocking the Trx system prevents NADPH from being used for cystine reduction, yielding effects similar to directly reducing NADPH. We collectively refer to these two strategies as reducing “available NADPH” (Figure 1).

The first criterion—enhancing cystine intake provides a strategy to intensify disulfidptosis.^3^ High expression of SLC7A11 or the provision of excess cystine can both enhance the occurrence of disulfidptosis. The details of the third condition remain unclear at this time. In practice, inducing disulfidptosis by increasing Rac‐WRC‐cytoskeletal proteins is not feasible, as these proteins are already abundantly expressed in cells. Further upregulation would likely be insignificant and could even promote tumor growth.^54^, ^55^ The primary purpose of elucidating the role of Rac‐WRC‐Arp2/3‐cytoskeletal proteins in disulfidptosis is to stratify patients and identify subtypes that are sensitive to disulfidptosis.

**FIGURE 2:**
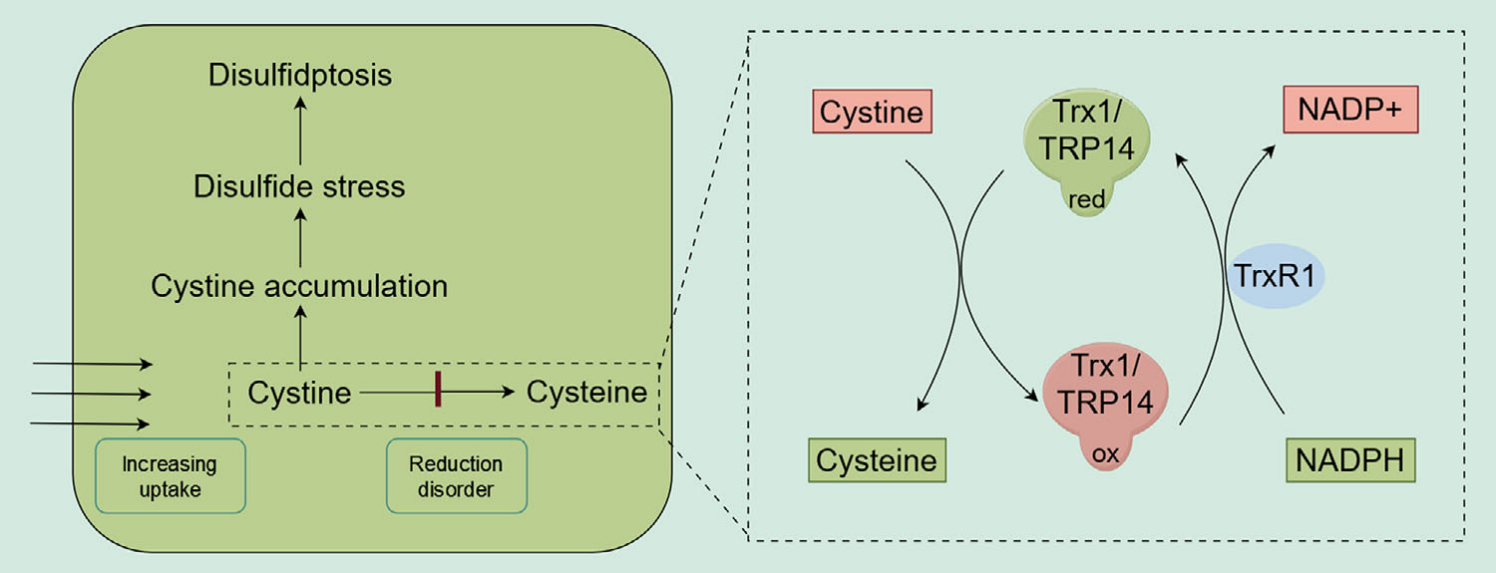
Pathways inducing disulfidptosis. When cells uptake large amounts of cystine while simultaneously blocking its reduction pathway, cystine accumulates, leading to disulfide stress and subsequently inducing disulfidptosis. The conversion of cystine to cysteine requires the thioredoxin (Trx) system, which utilizes NADPH as a substrate for the reduction reaction. Therefore, inhibiting the Trx system (with currently identified relevant proteins including Trx1, TrxR1, and TRP14) or directly reducing NADPH supply can induce disulfidptosis.

Therefore, after identifying sensitive subtypes, methods to induce disulfidptosis mainly involve two approaches: accelerating cystine uptake or hindering the conversion of cystine to cysteine (Figure 2). The latter can be achieved through two approaches: First, by inhibiting proteases involved in this conversion, such as TrxR1. Second, by directly reducing cytosolic NADPH levels, which can be accomplished by either blocking its production or increasing its consumption (Figure 2).

### 3.1 Increased cystine uptake

Cystine**/**Cysteine is crucial for maintaining cellular redox homeostasis, serving as an essential component of major antioxidants like GSH. To ensure a steady supply of cystine/cysteine, cells have developed several mechanisms including: (1) In certain cells and tissues, cysteine is synthesized from homocysteine via cystathionine γ‐lyase (CSE), the only known cysteine biosynthetic enzyme in mammals.^11^ (2) Intracellular degradation of proteins containing Cystine**/**Cysteine. Lysosomes, which are membrane‐bound organelles within cells, break down specific nutrients such as fats, proteins, and carbohydrates and export their breakdown products, like cystine.^56^ Given the oxidative environment within lysosomes, cystine is not reduced to its more soluble form, cysteine. If cystine cannot be effectively expelled from lysosomes into the cytosol's reducing environment, it cannot dissolve and instead forms crystals, slowly causing damage to various organs and tissues, which is how cystinosis develops.^57^ (3) Primary uptake from the extracellular environment as cystine, which is then reduced to cysteine inside the cell.^12^ This method is most common among tumor cells.^28^ Importantly, cells can also directly absorb cysteine, but due to the oxidative nature of the extracellular environment, cysteine is unstable and rapidly oxidizes back to cystine, making extracellular cysteine concentrations significantly lower than those of cystine. As a result, most cells heavily depend on SLC7A11 to obtain extracellular cystine to meet their internal Cystine**/**Cysteine needs.^5^, ^28^

Given these three primary sources of Cystine**/**Cysteine, methods to induce disulfidptosis through the manipulation of cystine are limited. De novo synthesis of cysteine does not involve the consumption of NADPH to convert cystine.^11^ Lysosomal cystine appears to contribute to disulfidptosis to some extent. Inducing rapid lysosomal cystine release might increase cytosolic cystine levels, consuming NADPH and thereby facilitating disulfidptosis.^58^ Apart from the inherent vulnerability of tumors with high SLC7A11 expression to undergo disulfidptosis, additional supplementation of cystine has been shown in vitro to induce disulfidptosis in tumors with low SLC7A11 expression.^3^ However, applying this approach in vivo may require further exploration, as cystine is poorly soluble in neutral environments and does not readily accumulate in tumor sites.^59^

### 3.2 Inhibition of the thioredoxin system

The reduction of cystine may depend on several enzyme systems, whose roles have often been overlooked. Some studies suggest that glutathione (GSH) can reduce cytoplasmic cystine.^11^ However, GSH primarily functions in scavenging reactive oxygen species (ROS), and its reduction of l‐cystine is too slow to provide the necessary levels of cysteine within the cell.^11^, ^22^ The thioredoxin (Trx) system plays a significant role in this process.^22^, ^23^ The Trx system is composed of different thioredoxins (Trx) and thioredoxin reductases (TrxR), with TrxR utilizing NADPH to reduce the active site disulfides in its Trx substrates back to dithiols.^60^ There are two subtypes of Trx: cytoplasmic Trx1 and mitochondrial Trx2.^27^ Research has shown that Trx1 significantly contributes to the reduction of l‐cystine within cells.^11^, ^22^ A recent study identified TRP14, a Trx‐like protein, as a rate‐limiting enzyme in cystine reduction.^22^ Knockdown of TRP14 leads to elevated cystine levels, yet paradoxically, this knockdown alleviated pancreatitis,^22^ even though other studies have suggested cystine accumulation and disulfide stress occur during pancreatitis.^26^ Therefore, more in‐depth research on TRP14 is needed.

TrxR1 converts oxidized Trx1 and TRP14 back to their reduced forms by consuming NADPH (Figure 2). Inhibiting TrxR1 can disrupt the reduction of cystine to cysteine, potentially leading to disulfidptosis in cells with high SLC7A11 expression, such as osteoclast precursors.^21^

It's important to note that the Trx system not only plays a role in reducing cystine but is also crucial for clearing disulfide bonds between proteins.^49^ Therefore, inhibiting the Trx system could both increase cystine accumulation and exacerbate the formation of interprotein disulfide bonds.

In summary, inhibiting the Trx system might trigger disulfidptosis even in cases where NADPH levels are sufficient, a concept often overlooked in previous research and reviews. Currently identified targets include Trx1, TRP14, and TrxR, but further studies are needed to determine whether other proteins are involved. PX‐12, the only Trx1 inhibitor to enter clinical trials, has shown in vitro efficacy against various tumors. A Phase I clinical trial indicated that PX‐12 could reduce Trx1 levels in patients, potentially offering therapeutic benefits for patients with advanced cancers.^61^ In addition to PX‐12, PMX464 is another known Trx inhibitor.^62^ TrxR1 inhibitors, such as auranofin,^63^, ^64^, ^65^ WZ26,^66^, ^67^ and selenocystine,^63^ can increase ROS levels and induce apoptosis in cancer cells. However, clinical trials for TrxR1 inhibitors are still lacking. Future research should focus on the role of these inhibitors in inducing disulfidptosis. The significance of TRP14 has only recently been recognized, and corresponding inhibitors have yet to be developed^22^ (Table 1).

**TABLE 1:** The preclinical and clinical studies with inhibitors targeting the Trx system in cancers.

Target	Inhibitor	Tumor type	Clinical trial ID	Phase
Trx1	PX‐12	Advanced solid tumors^61^	NCT00736372	Phase 1
	PMX464	colorectal cancer^62^		
	Selenocystine	lung cancer^63^		
	Auranofin	breast cancer,^64^ lung cancer^65^		
	WZ26	cholangiocarcinoma,^66^ colon cancer^67^		
TRP14	No	No		

### 3.3 Reducing cytosolic NADPH production

Directly reducing NADPH supply to inhibit the conversion of cystine to cysteine is another effective method to induce disulfidptosis. Current research primarily focuses on this approach.^3^, ^24^ However, cancer cells have evolved various mechanisms to generate NADPH, necessitating a thorough understanding of NADPH sources to selectively target the specific NADPH production pathways in particular tumors.

**FIGURE 3:**
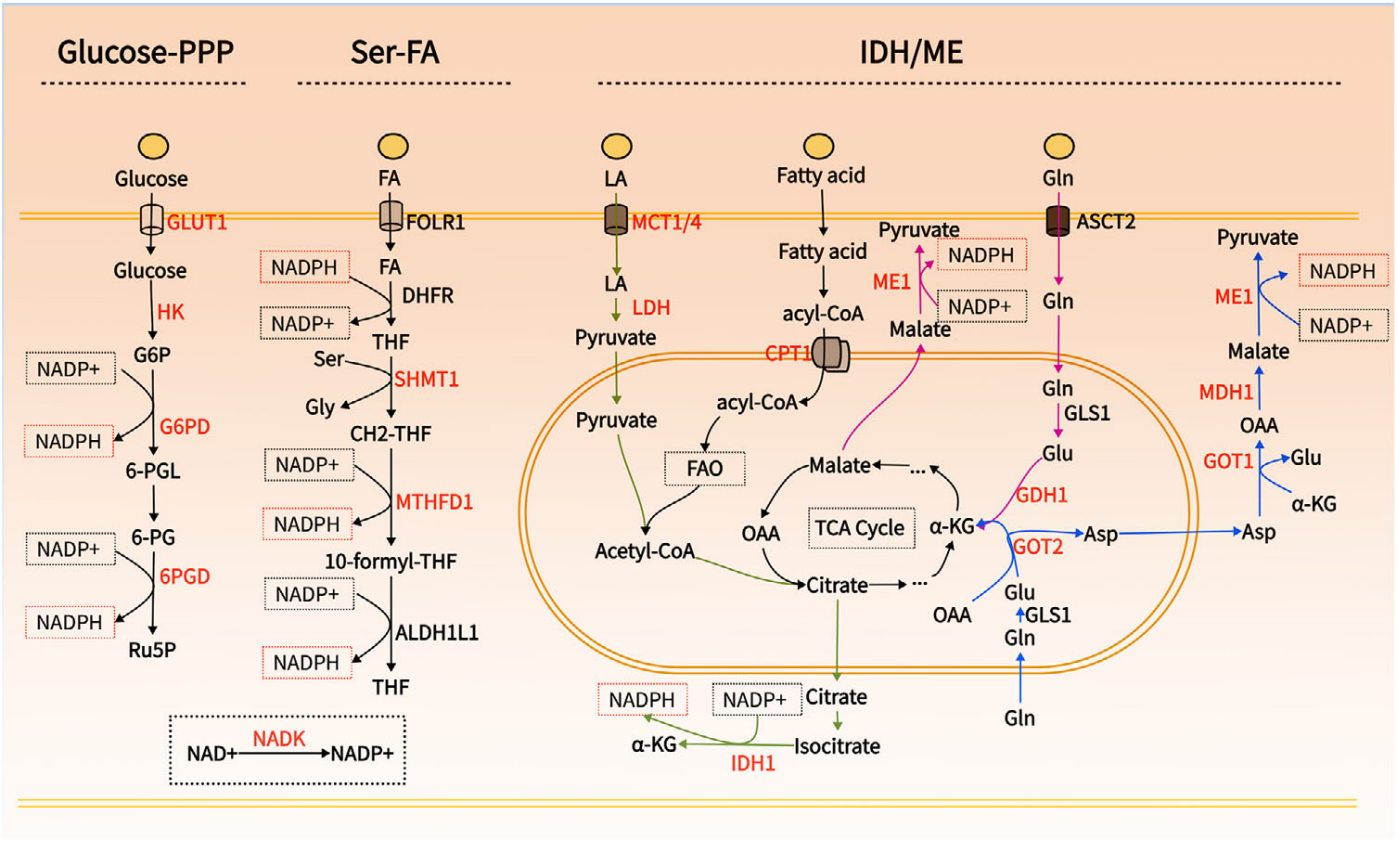
Pathways of cytoplasmic NADPH production in cancer. Tumors rely on the pentose phosphate pathway (PPP), serine‐folate metabolism, and lactate, glutamine, and fatty acid metabolism mediated by IDH and ME for NADPH production. NADK is the sole enzyme responsible for producing NADP+. While most cells depend on the PPP for NADPH production, tumor cells often face a nutrient‐deprived microenvironment where alternative pathways compensate during glucose scarcity. In some cancers, folate metabolism can contribute to NADPH production comparably to PPP. Additionally, tumors frequently exist in high lactate environments with enhanced fatty acid oxidation; both can produce acetyl‐CoA entering the tricarboxylic acid (TCA) cycle. Lactate metabolism exits the mitochondria as citrate under the action of IDH1 to produce NADPH. It is currently unclear whether fatty acid oxidation contributes to NADPH production via the citrate or malate pathways, warranting further research. In most cells, glutamine metabolism to glutamate by GDH1 generates α‐KG that enters the TCA cycle, exiting the mitochondria as citrate (in high lactate conditions) or malate (in low lactate conditions) to produce NADPH in the cytoplasm. In KRAS‐mutated tumors, glutamate is converted to aspartate by GOT2 in mitochondria; aspartate exits to the cytoplasm where it is transformed into malate by GOT1 and MDH1, with malic enzyme then generating NADPH.

**FIGURE 4:**
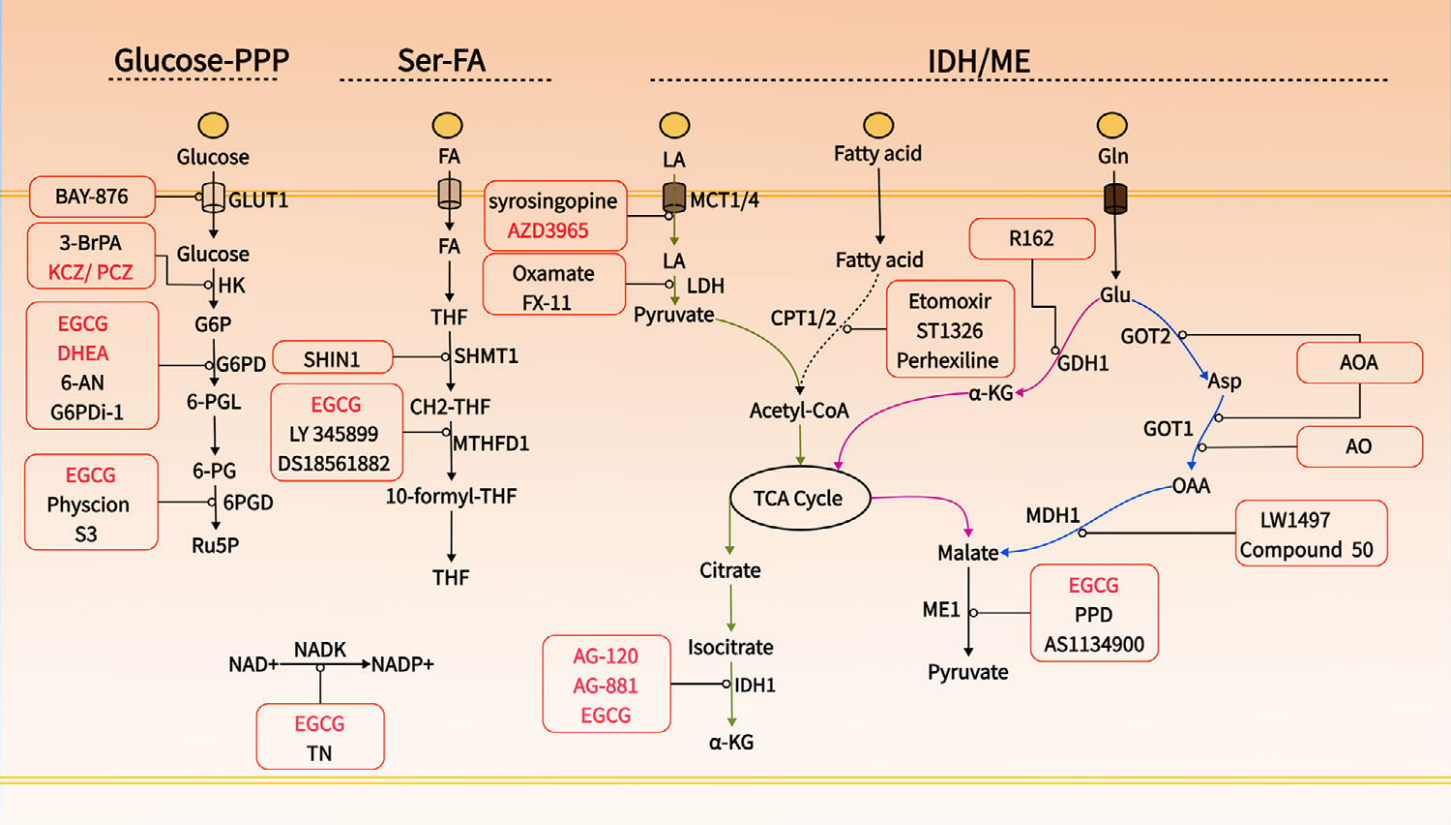
Therapeutic implications for targeting NADPH metabolism. Numerous inhibitors targeting key enzymes involved in NADPH production pathways have been found to deplete the NADPH pool, thereby attenuating tumor initiation and progression. (Drugs that have entered clinical trials are represented in red font, while those in preclinical experiments are shown in black font.) S3, Physcion derivative S3.

NADPH serves as a crucial electron donor in biological organisms, supplying the necessary reducing power for synthetic metabolic reactions and redox balance maintenance.^5^ Notably, NADPH is compartmentalized, with cytoplasmic and mitochondrial pools remaining separate.^5^, ^68^ Given that the reduction of cystine relies on cytoplasmic NADPH, this discussion focuses on the metabolism of cytoplasmic NADPH. Cancer's unique metabolic characteristics involve various pathways contributing to NADPH production, including the PPP, folate metabolism, IDH/ME‐mediated lactate, glutamine, and fatty acid metabolism^5^, ^69^ (Figure 3). These pathways present multiple targets for reducing NADPH production. Hereafter, we will explore strategies to decrease cytoplasmic NADPH production from various perspectives to potentially induce disulfidoptosis (Table 2, Figure 4).

**TABLE 2:** The preclinical and clinical studies with inhibitors targeting NADPH metabolism in cancers.

Target	Inhibitor	Tumor type	Clinical trial ID	Phase
NADK	Thionicotinamide	Lymphom^75^		
NADK/G6PD/ 6PGD/IDH1/ ME1/MTHFD1/2	EGCG	Colorectal cancer^76^	NCT02891538	Phase 1
GLUT1/3	BAY‐876	Breast cancer^86^/Colon cancer^87^		
HK	3‐Bromopyruvic acid	Neuroblastoma^91^		
	Ketoconazole and posaconazole	Glioblastoma^92^	NCT03763396	Phase 1
			NCT04869449	Phase 1
G6PD	DHEA	Breast cancer^94^	NCT01376349	Phase 3
	6‐aminonicotinamide	Cervical cancer^98^		
	G6PDi‐1	No		
6PGD	Physcion /S3	Prostate cancer^100^/lung cancer^101^		
SHMT1/2	SHIN1	Lung cancer^113^/ rhabdomyosarcoma^114^		
MTHFD1/2	LY 345899	Colorectal cancer^115^		
	DS18561882	Breast cancer^116^/prostate cancer^117^		
ME1	Piperazine‐1‐pyrrolidine‐2,5‐dione scaffold	Colorectal cancer^126^		
	AS1134900	No		
mIDH	AG‐120	Cholangiocarcinoma^134^	NCT02989857	Phase3
		Acute myeloid leukemia^136^	NCT03839771	Phase3
	AG‐881	Glioma^138^	NCT04164901	Phase3
MCT1/4	Syrosingopine	breast cancer^145^		
MCT1	AZD3965	Burkitt lymphoma^146^	NCT01791595	Phase1
LDHA	Oxamate	Glioblastomas^147^/medulloblastomas^148^		
	FX11	Lymphoma^149^/pancreatic cancer^149^		
GDH1	R162	Lung cancer^152^/breast cancer^155^		
GOT1/2	Aminooxyacetic acid	Breast cancer^158^		
GOT1	AO	Pancreatic ductal adenocarcinoma^159^		
MDH1/2	LW1497	Lung cancer^160^		
	Compound 50	Lung cancer^161^		
CPT1	Etomoxir	Glioblastoma^167^		
	ST1326	Leukemia^168^		
CPT2	Perhexiline	Gastrointestinal cancers^170^		

*Note*: Drugs in red font represent the relevant drug entering clinical trials, and drugs in black font represent preclinical trials.

#### 3.3.1 NAD+ kinase

NADPH is regenerated by the reduction of NADP+ via various enzymatic systems within the cell.^5^ The sole known enzymatic pathway for synthesizing NADP+ is catalyzed by NAD+ kinase (NADK), which exists in two isoforms: cytosolic NADK and mitochondrial NADK2.^70^ NADK facilitates the conversion of NAD+ to NADP+ by transferring a phosphate group from a nucleotide donor, typically ATP, to the 2′ position of NAD+.^71^ In a study on pancreatic cancer, it was demonstrated that cytosolic NADK, rather than mitochondrial NADK2, is the enzyme responsible for the increased formation of nascent NADP+. Knockdown of NADK reduced the total NADP+/NADPH pool in pancreatic and breast cancer cells, inhibiting tumor progression and metastasis.^72^ The role of cytosolic NADK suggests that inhibiting NADK holds significant potential for inducing disulfidptosis, particularly in tumors that express high levels of both SLC7A11 and NADK, such as breast and pancreatic cancers.^72^, ^73^ NADK activity is inhibited by thionicotinamide adenine dinucleotide and thionicotinamide adenine dinucleotide phosphate, both of which are generated from the prodrug thionicotinamide within the cell and act as inhibitors by targeting the NAD binding site of NADK.^74^, ^75^ Additionally, Epigallocatechin gallate (EGCG) has shown noncompetitive inhibition of NADK.^76^

#### 3.3.2 Glucose‐PPP

The PPP serves as the primary route for NADPH production in most cells.^51^ This pathway starts with glucose‐6‐phosphate, which is formed when glucose, taken up by GLUT, is converted by hexokinase (HK).^77^, ^78^ Both GLUT and HK are critical for NADPH production and tumor progression.^79^ Cancer cells require increased glucose uptake to meet their heightened energy, biosynthetic, and redox demands, resulting in the overexpression of GLUT.^80^ Among the twelve identified GLUT proteins, GLUT1 and GLUT3 are particularly prevalent and crucial for tumor metabolic activation and glycolysis, contributing to malignant characteristics and resistance to radiotherapy and chemotherapy.^81^ Inhibiting GLUT1/3 depletes cellular NADPH, leading to cell death via ferroptosis or disulfidptosis.^3^ In mammals, five HK isozymes (HK1, HK2, HK3, glucokinase, and hexokinase domain‐containing 1) have been identified, among which, HK2 is frequently upregulated in cancer and is crucial for maintaining NADPH levels, making it a promising target in the development of anticancer strategies.^82^, ^83^ Additionally, HK2 facilitates tumor angiogenesis and immune evasion.^84^ Inhibiting HK2 has been effective in eliminating chemotherapy‐resistant malignant tumors.^85^

Inhibiting GLUT and HK may effectively induce disulfidptosis, offering a promising approach for targeting cancer cells.^3^ Inhibitors targeting GLUT, such as BAY‐876 and 2‐deoxy‐d‐glucose (2‐DG), have shown potent antitumor effects.^80^, ^86^, ^87^ BAY‐876 effectively reduces intracellular NADPH and promotes disulfidptosis in tumor cells expressing high levels of SLC7A11.^3^ Although 2‐DG, a glucose analog, competes with GLUT1 and inhibits HK, it does not participate in glycolysis. However, 2‐DG can engage in the initial reactions of the PPP, where it contributes to NADPH production, potentially limiting its use as a disulfidptosis inducer.^3^ Furthermore, direct glucose consumption can also decrease intracellular levels of NADPH. Catalyzed by glucose oxidase (GOx), significant quantities of oxygen and glucose are rapidly used, producing H_2_O_2_ and D‐gluconic acid. Given its effectiveness in glucose depletion, GOx has been extensively employed in therapeutic strategies aimed at starving tumors.^88^, ^89^ The production of H2O2 further depletes NADPH, thereby endowing GOx with potent disulfidptosis‐inducing capabilities.^90^

Beyond 2‐DG, HK inhibitors such as 3‐bromopyruvic acid (3‐BrPA) have shown distinct anti‐tumor effects and enhance the efficacy of chemotherapy.^79^, ^91^ The only HK inhibitors currently undergoing clinical trials are the azole drugs ketoconazole (KCZ) and posaconazole (PCZ), which are used clinically as antifungal agents.^92^ These inhibitors exert their effect by targeting pathways regulated by HK2 are now in Phase I clinical trials (NCT03763396/NCT04869449).

When glucose‐6‐phosphate is produced, the PPP processes glucose‐6‐phosphate in two phases^51^: an oxidative phase that produces ribose phosphate, NADPH, and CO_2_, followed by a nonoxidative phase that generates fructose‐6‐phosphate and glyceraldehyde‐3‐phosphate, with all reactions taking place in the cytoplasm. The production of NADPH in the first phase involves three irreversible enzyme‐catalyzed reactions: (1) glucose‐6‐phosphate is oxidized to 6‐phosphogluconolactone by glucose‐6‐phosphate dehydrogenase (G6PD), during which NADP+ is reduced to NADPH; (2) 6‐phosphogluconolactone is hydrolyzed to 6‐phosphogluconate by lactonase; (3) 6‐phosphogluconate dehydrogenase (PGD or 6PGD) catalyzes the oxidative decarboxylation of 6‐phosphogluconate to ribulose‐5‐phosphate, simultaneously producing NADPH. Lactonase, due to its non‐specificity, has received less attention. However, increasing studies show that activities of G6PD and 6PGD are elevated in various cancers such as bladder, breast, prostate, and gastric cancers compared with normal tissues, indicating poor prognosis and playing crucial roles in tumor development and chemotherapy resistance.^93^, ^94^ Deficiency in G6PD or 6PGD can significantly reduce NADPH levels and enhance the apoptosis induced by chemotherapy drugs through redox modulation.^95^ Knockdown of G6PD promotes glucose restriction‐induced cell death, whereas its overexpression mitigates this effect, demonstrating the potential of G6PD and 6PGD in reducing NADPH levels and thereby inducing disulfidptosis.^12^

Antagonists of G6PD are promising clinical therapeutic drugs.^96^ Current developments include dehydroepiandrosterone (DHEA), 6‐aminonicotinamide, G6PDi‐1, and EGCG.^95^, ^96^, ^97^, ^98^ Clinical trials have assessed the effects of DHEA on inhibiting NADPH production and inducing cell death in conditions like synovial sarcoma and breast cancer, now progressing to Phase 1 and Phase 3 trials (NCT02683148, NCT01376349). EGCG, acting as a competitive inhibitor of NADP(+) for enzymes such as G6PD, IDH, ME, MTHFD1, ALDH1L1, and 6PGD, is currently under Phase 1 trials to evaluate its anticancer effects (NCT02891538).^99^ Additionally, EGCG can be used as a protective drug after radiotherapy and chemotherapy and has entered the phase II clinical trial (NCT02580279) for breast neoplasms and lung neoplasms (NCT02577393). Besides EGCG, 6PGD inhibitors like Physcion and its derivative S3 have shown specific inhibitory effects on 6PGD and promising anticancer efficacy.^100^, ^101^

#### 3.3.3 Serine‐folate mediated one‐carbon metabolism

Folate metabolism has long been recognized as critical for proliferating cells due to its role in providing one‐carbon units, which is essential for the synthesis of thymidylate, purines, and methionine.^102^, ^103^ However, another pivotal function of this pathway is its ability to generate reducing power.^104^ It is estimated that up to 40% of NADPH production in certain cells originates from serine‐tetrahydrofolate (THF) mediated one‐carbon metabolism.^105^ The process of folate metabolism begins with NADPH consumption to produce THF, catalyzed by dihydrofolate reductase (DHFR). Subsequently, THF undergoes a reversible reaction with serine, catalyzed by serine hydroxy methyltransferase 1 (SHMT1), to produce glycine and methylene‐THF. Methylene‐THF is then converted into NADPH and 10‐formyl‐THF by methylene tetrahydrofolate dehydrogenase 1/2 (MTHFD1/2), and further processed into a second NADPH molecule and back to THF by aldehyde dehydrogenase family 1 member L1/2 (ALDH1L1/2)^106^ (Figure 3).

Folate cycling occurs both in the cytoplasm and mitochondria, with MTHFD1 and ALDH1L1 located in the cytoplasm, and MTHFD2 and ALDH1L2 found in the mitochondria.^5^ Cytoplasmic ALDH1L1, which is less expressed in cancer cells, contributes minimally to cytosolic NADPH levels. Conversely, ALDH1L1 has been found to play a surprisingly tumor‐suppressive role rather than promoting cancer.^107^ In contrast, MTHFD1 is upregulated in various cancers and associated with poor prognosis.^108^, ^109^ Knockdown of MTHFD1 significantly reduces cellular NADPH levels.^105^, ^108^ Given these findings, MTHFD1 may serve as a critical target for inducing disulfidptosis. Additionally, serine metabolism mediated by SHMT1 provides the serine substrate for folate metabolism, and genetic deletion of SHMT1 significantly reduces NADPH levels, leading to apoptosis in lung cancer cells induced by uracil misincorporation.^105^ However, SHMT1 suppresses HCC metastasis by inhibiting NOX1‐mediated ROS production, highlighting the complexity of its functions.^110^

Anyway, targeting SHMT1‐MTHFD1 effectively reduces NADPH levels, underscoring its potential for inducing disulfidptosis. It is noteworthy that THF production is a NADPH‐consuming process, creating a balance where cells may burn one‐carbon units to produce NADPH if the supply exceeds NADPH availability.^105^ Therefore, targeting folate receptors (FOLR1, the main protein mediating folic acid absorption) and DHFR, may not be suitable for reducing NADPH levels. However, FOLR1 is overexpressed in various cancers and could serve as a target for drug delivery, offering an intriguing approach for delivering drugs targeting SHMT1‐MTHFD1.^111^, ^112^ SHIN1, an inhibitor of SHMT1/2, shows anticancer activity and may be effective in inducing disulfidptosis.^113^, ^114^ LY 345899, a folate analog and an inhibitor of both MTHFD1 (DC301) and MTHFD2.^115^ DS18561882, which inhibits both MTHFD1 and MTHFD2 but is more potent against MTHFD2, are also noteworthy in this context.^116^, ^117^

#### 3.3.4 Malic enzyme/Isocitrate dehydrogenase

ME and IDH within the cytoplasm catalyze the malate and isocitrate to generate NADPH, respectively.^53^, ^69^ The dependence of ME/IDH on lactate, glutamine, and fatty acid metabolism for NADPH production varies depending on the type of tumor and the state of the microenvironment.^53^, ^69^, ^118^ The generation of NADPH by ME/IDH is essential for cellular survival, particularly when the PPP pathway is inhibited.^53^

ME oxidatively decarboxylates malate into pyruvate, linking glycolysis and the Krebs cycle, and induces biosynthetic metabolism while concurrently producing NADPH.^119^ Flux analysis indicates that in some cells, ME's direct contribution to NADPH production is comparable to that of PPP.^69^, ^105^, ^120^ The ME family consists of three isoforms: ME1 in the cytoplasm, and ME2 and ME3 in the mitochondria. ME1 and ME3 utilize NADP+, whereas ME2 can use either NAD+ or NADP+ for catalytic activity. ME1 and ME2 appear to be the primary isoforms, as ME3 is nearly undetectable in many assessed mammalian cells.^121^ Overexpression of ME1 has been significantly associated with poor prognosis in cancers such as gastric, oral squamous cell, breast, and lung cancers.^121^, ^122^ Silencing ME1 substantially lowers NADPH levels, increases ROS levels, and ultimately induces cell death under oxidative stress conditions like glucose starvation or hypoxia, raising the question of whether this type of death is related to disulfidptosis.^122^ Moreover, a study shows that a hydride transfer complex composed of malate dehydrogenase 1, ME1, and cytoplasmic pyruvate carboxylase, by transferring the reducing equivalents from NADH to NADP+, reprograms NAD metabolism to enhance NADPH production and inhibit senescence.^123^ Interestingly, there is direct cross‐talk between ME1 and PPP components; ME1 increases the binding ability of PGD to 6‐phosphogluconate, enhancing NADPH production.^124^ This suggests that targeting both ME1 and the PPP pathway could be more effective in inhibiting NADPH production and beneficial for inducing disulfidptosis. There is still a lack of effective, recognized inhibitors of ME1. Known molecules inhibiting ME1 activity are those based on the piperazine‐1‐pyrrolidine‐2,5‐dione scaffold.^125^, ^126^ Other ME1 inhibitors include AS1134900.^127^

IDH catalyzes the oxidative decarboxylation of isocitrate to α‐ketoglutarate (α‐KG) and NADPH. There are three isoforms of IDH: IDH1 is located in the cytoplasm and peroxisomes, while IDH2/3 are mainly in the mitochondria. IDH1 and IDH2 convert NADP+ to NADPH, whereas IDH3 converts NAD+ to NADH and participates only in the citric acid cycle.^128^ IDH1 is overexpressed in various cancers and is closely associated with poor prognosis in non‐small cell lung cancer, pancreatic ductal adenocarcinoma (PDAC), and several hematological malignancies.^129^, ^130^, ^131^ The upregulation of IDH1 may represent a common metabolic adaptation to reduce oxidative stress and support macromolecule synthesis, thus promoting tumor growth.^131^ Compared with normal brain tissue, IDH1 is the most upregulated enzyme producing NADPH in glioblastoma, and its mRNA and protein expression increase postradiation, indicating its role in the cellular response to radiation.^132^ Interestingly, mutations in IDH1 and IDH2 are prevalent in various malignancies, including gliomas, AML, vascular immunoblastic lymphoma, chondrosarcoma, and melanoma.^133^, ^134^ Mutated IDH1 and IDH2 (mIDH1/2) proteins gain the ability to reduce α‐KG to an unusual metabolite, 2‐hydroxyglutarate (2‐HG), while consuming NADPH rapidly.^135^ Given the role of IDH in NADPH production, targeting IDH1 may aid in inducing disulfidptosis, and the susceptibility of IDH‐mutated individuals to disulfidptosis warrants further investigation. Significant advances have been made with inhibitors for mIDH, and the mIDH1 inhibitor—AG‐120 (Ivosidenib)—targeting mutated IDH1 has been FDA‐approved for treating acute myeloid leukemia.^136^ However, effective inhibitors for wild‐type IDH1‐producing NADPH are lacking, although some developed mIDH1 inhibitors like AG‐120 and AG‐881 show inhibitory activity against the wild‐type enzyme at higher concentrations.^137^, ^138^

#### 3.3.5 Lactate

Lactate's contribution to NADPH production has been historically underestimated, yet recent findings reveal that lactate facilitates NADPH generation through at least two mechanisms. First, as a metabolite, it redirects glucose metabolism toward the pentose phosphate pathway, thereby elevating NADPH levels.^139^ Second, lactate directly participates in the tricarboxylic acid (TCA) cycle through the action of IDH to produce NADPH.^53^ Research indicates that lactate is crucial for supplementing NADPH during glucose deficiency; the addition of exogenous lactate can partially offset the decrease in NADPH caused by glucose shortage.^53^ This is because cancer cells utilize lactate to synthesize pyruvate, which then enters the TCA cycle as acetyl‐CoA and subsequently forms citrate. Once citrate is transported to the cytoplasm, it is converted to isocitrate, which under the influence of IDH, produces α‐KG and NADPH.^53^ In vivo, lactate within tumor cells is primarily produced by LDHA/B from pyruvate or taken up from the extracellular environment through MCT1 and MCT4 transporters.^140^ Although LDH and MCT are known to facilitate tumor progression through high expression in various cancers, direct reports on their roles in enhancing NADPH production are lacking. Studies have shown that MCT1‐mediated lactate uptake protects pancreatic adenocarcinoma cells from oxidative stress in the absence of glutamine.^141^ It is noteworthy that cancer cells may exhibit opposite functions of MCT in vitro (exporting lactate) and in vivo (primarily importing lactate), and MCT may display cellular heterogeneity within the same tumor.^142^ Nevertheless, given the crucial role of LDH and MCT in lactate metabolism, it is hypothesized that they might significantly influence NADPH production in certain cancers.

Strategies for regulating NADPH based on lactate metabolism include two approaches: (1) directly reducing lactate levels using lactate oxidase to break down lactate or inhibiting MCT1/MCT4 to limit lactate uptake; (2) Inhibiting intermediate reactions in lactate metabolism, such as LDH. Lactate oxidase, formulated as a nanomedicine, has shown potent effects in treating tumors alone or in combination with other drugs.^143^, ^144^ Furthermore, a variety of MCT inhibitors have been developed, particularly those targeting MCT1, which have made significant advancements. Syrosingopine, an antihypertensive drug, acts as a dual inhibitor of MCT1 and MCT4 and has demonstrated potent antitumor activity.^145^ AZD3965, which specifically inhibits MCT1, has effectively restrained the growth of advanced solid tumors and lymphomas and has completed a phase I clinical trial, demonstrating efficacy and safety^146^ (NCT01791595). Additionally, various LDH inhibitors have been developed for treating cancers with high LDH expression, including Oxamate^147^, ^148^ and FX11.^148^, ^149^

#### 3.3.6 Glutamine

Glutamine metabolism serves as a major source of carbon for the TCA cycle in proliferating cells and acts as a nitrogen donor for the biosynthesis of nucleotides, amino acids, and lipids.^150^ It is also crucial for maintaining NADPH levels.^53^ Glutamine is transported into cells mediated by ASCT2 and converted into glutamate in the mitochondria by glutaminase (GLS). In most tumor cells, glutamate is converted into the TCA cycle intermediate α‐KG by glutamate dehydrogenase (GDH) or the transaminases GOT2/GPT2.^150^ α‐KG can then produce citrate or malate through the TCA cycle, which exits the mitochondria and is converted into NADPH in the cytoplasm by IDH or ME.^53^ The conversion of α‐KG to either citrate or malate in the mitochondria is determined by lactate levels. Under high lactate conditions, acetyl‐CoA produced from lactate condenses with oxaloacetate derived from α‐KG to form citrate, which is then exported from the mitochondria. Conversely, when lactate levels are low, α‐KG is metabolized in the TCA cycle and exits the mitochondria as malate to produce NADPH.^53^

Targeting glutamine metabolism to reduce NADPH could potentially involve five key points: ASCT2, GLS, GDH, and GOT/GPT. It is important to note that cystine transport depends on the counter‐transport of glutamate; inhibiting GLS or ASCT2 may reduce intracellular glutamate levels thus inhibiting SLC7A11's uptake of cystine, hence GLS and ASCT2 are more inclined to induce ferroptosis rather than disulfidptosis (simultaneously reducing NADPH and cystine). Research has found that inhibiting GLS1 alleviates disulfidptosis.^4^ Conversely, inhibiting the further metabolism of glutamate may have the dual effect of enhancing cystine uptake and reducing NADPH.

In tumors, the conversion of glutamate to α‐KG is predominantly mediated by GDH, including GDH1 and GDH2, rather than by transaminases.^53^ GDH1, not GDH2, is highly expressed in most tumor samples and is associated with the progression stage of the tumors, including lung and colorectal cancer.^151^, ^152^ Depletion of GDH1 leads to redox imbalance and cellular toxicity, whereas its impact on the proliferation of normal cells is minimal.^153^ This highlights the safety and efficacy of GDH1 inhibition. Yang et al.^154^ reported that glioblastoma cells require GDH1 to maintain viability in conditions of limited glucose availability. Glucose deprivation activates GDH activity, and inhibition of GDH1 sensitizes glioblastoma cells to glucose withdrawal. This highlights the potential of combining GDH1 inhibition with glucose deprivation to aid in inducing disulfidptosis. R162 is an effective GDH1 inhibitor that can increase ROS levels, thereby inhibiting cancer cell proliferation.^153^, ^155^

Although transaminases GPT and GOT do not majorly impact the production of α‐KG entering the TCA cycle, certain cancer cells, such as those in PDAC, rely on a noncanonical pathway of glutamine metabolism in the cytosol under the regulation of oncogenic KRAS activation.^156^ GOT2 catalyzes the conversion of glutamate to aspartate, which is then transported to the cytosol and converted to oxaloacetate by GOT1. This is further converted to malate‐by‐malate dehydrogenase (MDH1) and subsequently oxidized to pyruvate by malic enzyme (ME1), producing NADPH.^156^ Targeting this cellular process for inducing disulfidptosis could involve inhibiting GOT2, GOT1, and MDH1. Knocking down GOT2 elevates ROS levels, leading to cellular senescence.^157^ Additionally, inhibiting cytosolic GOT1 can decrease oxaloacetate levels and lower the cellular NADPH/NADP+ and GSH/GSSG ratios.^156^ Those demonstrate the critical role of GOT1/2 in tumor maintenance of NADPH. Aminooxyacetic acid is a pan‐transaminase inhibitor active against GOT1/GPT2, showing inhibitory effects on various tumors.^158^ Developing specific GOT inhibitors remains challenging, but Aspulvinone O (AO) has been found to be an effective biological inhibitor of GOT1, sensitizing PDAC cells to oxidative stress and inhibiting cell proliferation.^159^

MDH exists in two isoforms, MDH1 in the cytosol and MDH2 in the mitochondrial matrix, with MDH2 involved in the TCA cycle.^156^ MDH1 is upregulated in various tumors, and inhibiting MDH1 impedes glutamine metabolism, making PDAC cells sensitive to oxidative stress. The addition of exogenous malate can protect cells with knocked‐down MDH1 from excessive ROS accumulation, highlighting the role of malate produced by MDH1 in contributing to NADPH production.^156^ LW1497 and Compound 50 are dual inhibitors of MDH1 and MDH2 with significant antitumor activity.^160^, ^161^ However, given that MDH2 primarily participates in the TCA cycle, there is a need to develop selective inhibitors for MDH1, which remains a challenge.

#### 3.3.7 Fatty acid oxidation

In addition to directly producing NADH and FADH2, which provide extra ATP crucial for cancer cell survival, fatty acid oxidation (FAO) also serves as an essential source of NADPH, particularly under metabolic stress in many cancers.^162^ For instance, in glioblastoma, even in the presence of glutamine and glucose, NADPH produced from fatty acid metabolism remains a key source.^118^ The rate‐limiting enzymes of the FAO pathway are the carnitine palmitoyltransferases (CPT1 and CPT2), located on the outer and inner mitochondrial membranes, respectively, coordinating the transport of acyl‐CoA into the mitochondria.^163^ CPT‐mediated FAO plays a critical role in maintaining NADPH homeostasis, aiding in cellular metastasis and chemoresistance in breast cancer cells and melanoma.^164^, ^165^ Inhibiting CPT impairs NADPH production, elevates ROS levels, leads to ATP depletion, and results in cell death.^118^ Interestingly, the type of cell death induced by this decrease in NADPH, which does not align with ferroptosis, could potentially be categorized as disulfidptosis—a hypothesis worth exploring.^118^

The pathway of NADPH production in FAO may parallel that of lactate, relying on the entry of acetyl‐CoA into the TCA cycle. Fatty acids are first activated to acyl‐CoA, transported into the mitochondria by CPT, and undergo β‐oxidation to produce acetyl‐CoA. This acetyl‐CoA then enters the TCA cycle, producing malate or citrate which, upon entering the cytosol, is converted to NADPH by either IDH1 or ME1.^69^, ^166^ However, it remains unclear whether IDH or ME plays a dominant role in this context.

Currently, inhibitors targeting CPT primarily focus on CPT1, including drugs like etomoxir and ST1326 (Tiglicarn).^118^, ^167^, ^168^ Etomoxir, a widely used FAO inhibitor, is applied in the development and treatment of type 2 diabetes and heart failure and has shown promising synergistic effects in chemotherapy and immunotherapy in various cancers such as gliomas and nasopharyngeal carcinoma.^167^, ^169^ Perhexiline, an inhibitor of CPT2, disrupts NADPH production and promotes apoptosis.^170^

#### 3.3.8 TCA cycle

The pathways for NADPH production through the metabolism of fatty acids, lactate, and glutamine involve the TCA cycle, producing citrate or malate which then enters the cytosol where it is converted into NADPH by the action of IDH1 and ME1. This raises the question of whether directly inhibiting the TCA cycle could impact NADPH production. The key enzymes in the TCA cycle are citrate synthase, isocitrate dehydrogenase (IDH3 in mitochondria, which produces NADH, not NADPH), and alpha‐ketoglutarate dehydrogenase.^171^ There is insufficient evidence to suggest that inhibiting these mitochondrial TCA cycle enzymes directly affects NADPH production.^172^ Conversely, some tumors show that a lack of citrate synthase promotes glycolysis and tumor progression.^173^ It does not seem wise to directly inhibit the TCA cycle to induce disulfidptosis for several reasons: First, the Warburg effect suggests that aerobic glycolysis mediated by the TCA cycle is already suppressed in tumors, enhancing anaerobic glycolysis, while normal cells rely on the TCA cycle for energy.^174^ Inhibiting the TCA cycle could potentially enhance anaerobic glycolysis, promoting tumor progression and adversely affecting normal cells.^172^ Second, direct inhibition of the TCA cycle is likely to impact ATP production rather than directly affecting NADPH.^172^ It could even lead to increased NADPH production by diverting metabolites to anaerobic metabolism and the PPP pathway, thus promoting tumor progression.^173^ Therefore, inhibiting the TCA cycle may not contribute significantly to inducing disulfidptosis in tumors, although further experimental evidence is needed.

### 3.4 Depletion of cytoplasmic NADPH

NADP(H) plays a pivotal role in cellular antioxidative functions and biosynthetic reactions.^69^ NADPH provides reductive equivalents to produce reduced forms of antioxidant molecules, which are intimately linked with the biological behavior of cancer cells. For instance, GSH reductase uses NADPH as an essential cofactor to convert GSSG to GSH, which then serves as a substrate for Glutathione peroxidase to reduce H_2_O_2_ and other peroxides to water or alcohols, thus deactivating ROS.^175^ Additionally, Trx is a redox‐regulating protein that modulates the activity and function of target proteins by reducing oxidized groups, such as disulfide bonds. Trx forms mixed disulfide bonds with target proteins and subsequently reduces their oxidized groups, a process that requires TrxR, which uses NADPH as an electron donor.^48^

Furthermore, NADPH serves as a key electron source for various reductive biosynthesis reactions, including the synthesis of fatty acids, amino acids, nucleotides, and steroids, which are essential for the rapid growth of tumor cells.^69^ However, strategies to enhance these synthetic reactions by consuming NADPH are generally counterproductive, as they tend to promote tumor progression.^176^, ^177^ The third function of NADPH involves the production of ROS through NADPH oxidase (NOX).^178^ ROS promote tumorigenesis by activating oncogenes such as Src and Ras and inactivating tumor suppressor proteins like TP53 and PTEN. Inhibiting NOX‐generated ROS can reduce the oncogenic effects across various cancers.^179^

Conversely, externally increasing ROS to deplete NADPH is a direct and effective approach, particularly through the addition of H_2_O_2_.^4^ As mentioned earlier, glucose oxidase and lactate oxidase, which produce H_2_O_2_ by oxidizing glucose or lactate, respectively, offer potential methods.^143^, ^180^ Certain chemotherapy drugs are known to increase ROS production, causing irreparable damage and cell death, including anthracyclines, cisplatin, bleomycin, doxorubicin, and arsenic trioxide.^181^ Photodynamic therapy uses photosensitizers, such as porphyrin compounds, to generate ROS under light exposure, inducing tumor cell death.^182^ However, whether solely increasing ROS or combining it with other strategies effectively induces disulfidptosis in vivo needs further validation, as it may lead to oxidative stress‐induced cell death rather than disulfidptosis. In conclusion, inducing disulfidptosis by depleting NADPH requires more research and should consider external approaches (external agents like H_2_O_2_ depleting NADPH) rather than solely relying on enhancing cellular metabolism.

## 4 DISULFIDPTOSIS AND IMMUNOTHERAPY

Regulated cell death frequently activates the innate and adaptive immune systems, particularly influencing macrophage and T‐cell immunity, providing a foundation for combination immunotherapy.^183^ For instance, inducing ferroptosis by releasing damage‐associated molecular patterns (DAMPs) enhances the activity of immune cells such as T cells and macrophages.^184^ The combination of immune checkpoint inhibitors with ferroptosis inducers can effectively amplify antitumor immunotherapy.^184^ Whether disulfidptosis similarly affects the immune microenvironment is an important question that needs to be addressed. This issue can be explored from two perspectives. First, from the outcome perspective: whether specific DAMPs are released during the disulfidptosis process is currently unexplored. Investigating the release of DAMPs during disulfidptosis could provide intriguing insights into potential synergies with immunotherapy. The second approach involves tailoring the immunotherapy strategy to the specific method used to induce disulfidptosis, thereby optimizing treatment efficacy. For example, in renal cancer, the expression of GLUT‐1 is negatively correlated with the number of CD8(+) T cells.^185^ Inhibiting GLUT1 expression results in an increased accumulation of CD4+ and CD8+ T cells within tumor tissues and a reduction in programmed death‐ligand 1 (PD‐L1).^185^ This suggests that inhibiting GLUT1‐mediated disulfidptosis could potentially be combined with immune checkpoint inhibitors to enhance therapeutic effectiveness.

### 4.1 Relationship between metabolites and tumor immunity

Previously, we discussed how cystine, glucose, folate, lactic acid, glutamine, and other substances are crucial raw materials for disulfidptosis. These substances also significantly impact the immune microenvironment, influencing various immune responses and interactions within the tumor milieu.^113^, ^158^, ^186^ For example, the limited availability of cystine in the tumor microenvironment (TME), largely due to its extensive consumption by tumor cells and immunosuppressive cells, has rendered it a significantly scarce amino acid within the TME. CD8+ T cells within the TME struggle to acquire sufficient cystine, leading to impaired function.^187^ The differential uptake of cystine by tumor cells and T cells suggests that increasing cystine levels in the TME could not only promote disulfidptosis in tumor cells but may also enhance T‐cell growth. Similarly, glucose also plays a critical role in tumor immunity. High tumor utilization of glucose leads to nutrient depletion in the TME and produces large amounts of lactate as a by‐product, thereby lowering the pH of the TME.^140^ This acidic environment impairs the cytolytic activity of T cells and the production of cytokines. Moreover, beyond its role in lowering pH, lactate is a metabolite with multiple effects on the immune cell population.^140^ It has been shown to polarize macrophages toward a tolerogenic M2 type and alter the metabolism of regulatory T cells (Tregs) to sustain their activity in a low‐glucose environment.^188^, ^189^

Glutamine metabolism enhances T‐cell activation and proliferation. Due to the heavy consumption of glutamine by tumors, a lack of glutamine in the TME might inhibit T‐cell activity.^190^ Combining treatment with the glutaminase inhibitor CB‐839 in lung adenocarcinoma can suppress CD8 T‐cell clonal expansion and activation.^190^ Enhanced fatty acid metabolism promotes invasive growth in glioblastoma (GBM) along with CD47‐mediated immune evasion. In GBM radioimmunotherapy, the FAO‐CD47 axis may improve GBM control by eliminating anti‐phagocytic tumor cells.^167^ Therefore, theoretically, inhibiting the concentration of glucose, lactate, glutamine, and fatty acids in the TME can affect tumor immunity. Reports on folate and the immune microenvironment are relatively scarce; however, studies have shown that MTHFD2 promotes basal and IFN‐γ‐stimulated PD‐L1 expression.^191^

Apart from the metabolites themselves, certain enzymes in these metabolic pathways can induce changes in the immune microenvironment through impacts on metabolites or other pathways.^186^ In the following discussion, we will explore the relationship between key enzymes involved in disulfidptosis, their impact on the immune microenvironment, and the implications for immunotherapy.

### 4.2 Trx1, TRP14, TrxR1 and immune cells

Studies have shown that blocking the Trx/TrxR system improves the tumor immune microenvironment by reversing the suppression of CD8+ T cells and NK cells within the tumor context.^192^ Targeting thioredoxin reductase 1 leads to disulfidptosis while simultaneously enhancing the efficacy of anti‐PD‐1 therapy in head and neck cancer.^193^ However, another study has identified Trx1 as crucial for maintaining redox homeostasis in T cells. Enhancing Trx1 in human T cells has been shown to improve the efficacy of CAR‐T cell therapy against solid tumors.^194^ This suggests that inhibiting Trx1 could also directly impair T‐cell function, which should be carefully considered when designing combination therapies. The role of TRP14 in immune function remains underexplored and warrants further investigation.

### 4.3 Glucose‐PPP and folate metabolism

In addition to GLUT, HK has been widely reported to influence the function of immune cells.^195^ HK2 induces the activation of NF‐κB, which enters the nucleus and promotes the expression of PD‐L1. Combining HK inhibitors with anti‐PD‐1 antibodies can eliminate tumor immune evasion and significantly enhance the antitumor effect of immune checkpoint blockade.^195^ In renal cancer, the upregulation of HK3 promotes M2 polarization of macrophages, which are known to support tumor growth.^196^ Overexpression of G6PD drives M2 macrophage polarization in TNBC cells by directly binding to phosphorylated STAT1 and upregulating the secretion of CCL2 and TGF‐β1.^197^ At the same time, G6PD expression is a prognostic marker detectable by immunohistochemistry and serum, and it negatively correlates with immune activity and PD‐L1 levels.^198^ PGD may also impact the use of immune checkpoint inhibitors. In liver cancer, high expression of PGD is associated with increased expression of immune checkpoints and a higher tumor mutation burden.^199^

Although studies have shown that folate metabolism affects the immune microenvironment, such as MTHFD2 promoting basal and IFN‐γ stimulated PD‐L1 expression, one role of MTHFD2 in human cancer development is promoting tumor immune escape.^191^ However, the impact of the SHMT1‐MTHFD1 axis on the immune microenvironment and immunotherapy still lacks clear research.

### 4.4 ME1/IDH1

The suppression of ME1 inhibits tumor growth, supported by substantial evidence, but its relationship with the immune microenvironment remains to be explored further. In T cells, overexpression of ME1 enhances the cytotoxicity and proliferation of effector CD8+ T cells, partly through the type I interferon pathway.^200^ This suggests that targeting ME1 should be approached with caution as it may suppress immune responses. In contrast, mutated IDH, particularly IDH1, is closely associated with immune suppression. Mutant IDH1 produces 2‐HG, which can be taken up by adjacent T cells via SLC13A3, inhibiting T‐cell activation.^135^ Combining αPD‐L1 immune checkpoint blockade with mutIDH1 inhibition significantly increases overall survival in glioma‐bearing mice with IDH1 mutation. Furthermore, this treatment strategy promotes the production of memory CD8+ T cells, leading to immune memory.^135^ Tumors with IDH1 mutation may be doubly sensitive to disulfidptosis and T‐cell immune therapy.

The key enzymes in lactate metabolism, LDH, and MCT, mainly influence the immune microenvironment by affecting lactate levels.^145^, ^201^, ^202^ For example, pharmacological inhibitors blocking the lactate transporters MCT1 and MCT4 reduce tumor acidification by preventing lactate release.^145^ Compared with checkpoint blockade alone, blocking MCT1/MCT4 in combination with checkpoint blockade enhances the response.^201^, ^203^ Downregulating the expression of LDHA, LDHB, and MCT inhibits the production and influx/efflux of lactate in cancer cells, promoting the repolarization of macrophages from the tumor‐supportive M2 phenotype to the tumor‐suppressive M1 phenotype.^204^

Regarding glutamine metabolism, the association of GOT1/2 and MDH1 with the immune microenvironment requires further experimental validation, although some database‐based studies suggest they may be related to T‐cell infiltration.^205^, ^206^

Studies indicate that the cytotoxicity of T cells is reciprocally influenced by fatty acid metabolism. Inhibiting CPT1 activity or expression makes cancer cells more susceptible to destruction by cytotoxic T lymphocytes.^163^ Furthermore, acetyl‐CoA from FAO upregulates CD47 transcription via the NF‐κB pathway; blocking CPT1 impairs tumor growth and reduces CD47's anti‐phagocytic effect.^167^ Etomoxir combined with anti‐CD47 antibodies synergizes with radiation to control regrowing tumors and enhance macrophage phagocytosis.^167^

**FIGURE 5:**
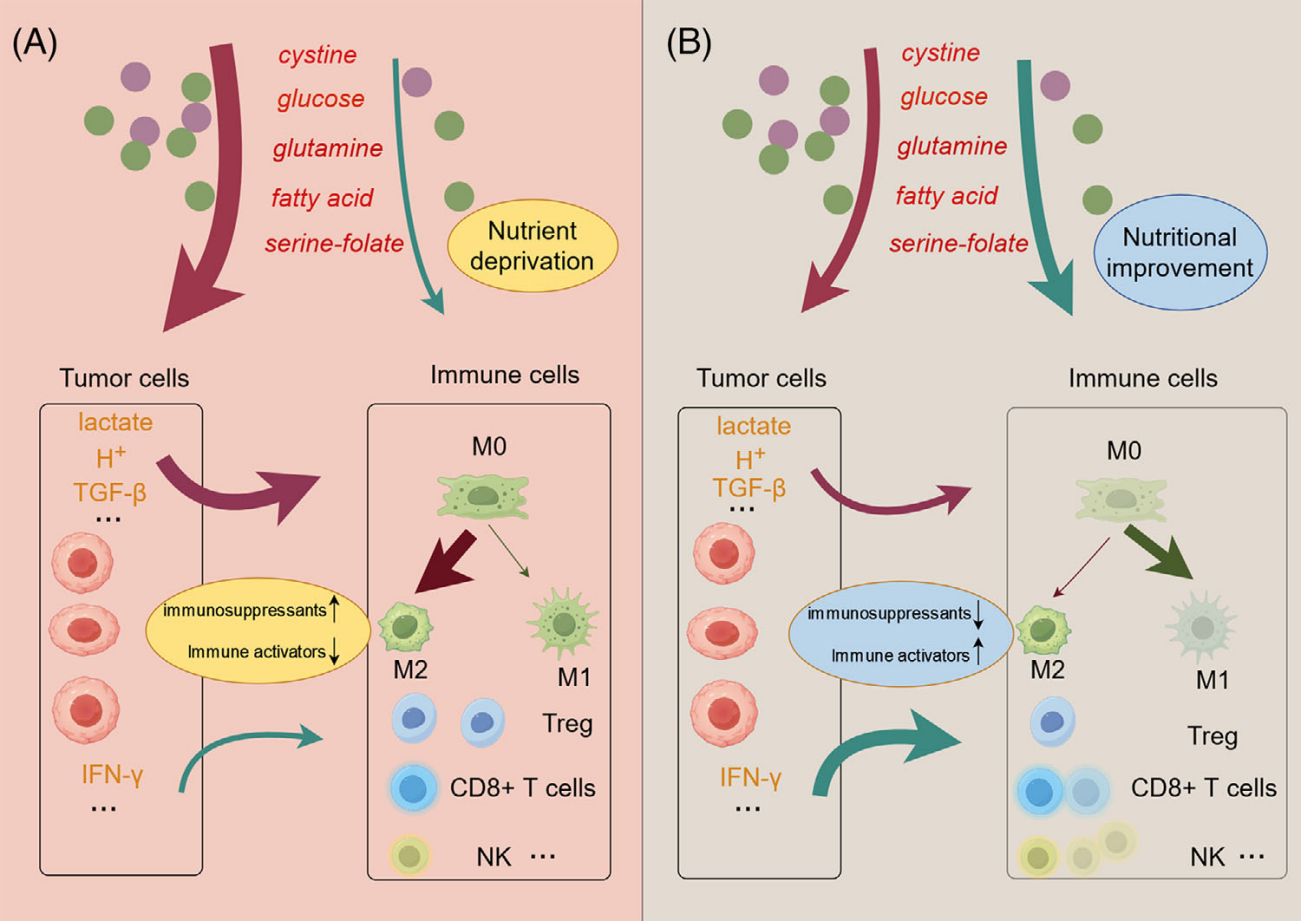
Disulfidptosis and immunotherapy. Cancer cells compete for nutrients, leading to a nutrient‐deprived state in immune cells, which impairs their antitumor immunity. Additionally, cancer cells can secrete immunosuppressive factors that enhance protumor immune cell functions while impairing those of anti‐tumor immune cells (A). Inducing disulfidptosis through specific methods can inhibit nutrient uptake by cancer cells and suppress their immunosuppressive activities, thereby enhancing the effectiveness of immunotherapy in cancer treatment (B). M0, M0 macrophages; M1, M1 macrophages; M2, M2 macrophages; NK, natural killer cells.

In summary, pathways that induce disulfidptosis impact the immune microenvironment, thereby influencing the effectiveness of immunotherapy. On one hand, antitumor immune cells within the TME often struggle to compete with tumor cells for nutrients. Inhibiting the uptake of glucose, glutamine, and other nutrients by tumor cells could help immune cells secure the resources they need to function effectively. On the other hand, tumor cells frequently secrete immunosuppressive factors, such as TGF‐β, which polarize macrophages toward a protumor phenotype. Certain methods of inducing disulfidptosis can also inhibit the secretion of these factors^197^ (Figure 5). Hence, future research should actively explore the effects of disulfidptosis on the immune microenvironment and investigate the potential of combining disulfidptosis induction with immunotherapy in cancer treatment.

## 5 CONCLUSION AND PROSPECTS

Disulfidptosis represents a metabolism‐related form of cell death, fundamentally driven by metabolic disturbances leading to excessive cystine accumulation and subsequent disulfide stress.^3^, ^207^ This type of cell death exhibits metabolic selectivity, particularly in cells with high SLC7A11 expression, making the induction of disulfidptosis a promising therapeutic strategy, especially in oncology. The ability to selectively induce disulfidptosis in tumor cells with minimal effects on normal cells, and potentially target cells resistant to traditional therapies, underlines its therapeutic potential.^207^

This review comprehensively outlines specific strategies for inducing disulfidptosis based on its underlying mechanisms. We propose targeting the Trx system or NADPH as viable approaches to induce disulfidptosis. Notably, Trx, TrxR, and TRP14 have emerged as promising targets, often overlooked, yet their roles require further elucidation. Additionally, understanding the involvement of other proteins in this process is crucial. NADPH has become a focal point in disulfidptosis research.^3^, ^208^ We have summarized the production of NADPH through three main pathways for the first time: the PPP, folate metabolism, and metabolism mediated by IDH and ME involving lactate, glutamine, and fatty acids.

Despite the comprehensive summary of potential disulfidptosis‐inducing strategies, significant challenges remain in translating these methods into viable clinical applications. First, some targets, such as TRP14, lack sufficient understanding and effective inhibitors, necessitating collective efforts to develop new compounds and determine their impact on disulfidptosis. Additionally, personalized medicine approaches must be developed to identify disulfidptosis‐sensitive subtypes (e.g., tumors with high SLC7A11 or WRC expression) and tailor specific induction strategies (e.g., using different inhibitors depending on the tumor's NADPH source). Combining multiple disulfidptosis induction pathways for synergistic effects should also be considered.

Exploring disulfidptosis in conjunction with other forms of cell death is also essential. Disulfidptosis and other cell death modalities are not mutually exclusive; rather, they can be complementary or even synergistic in antitumor strategies.^24^ The synergistic anti‐tumor effects of disulfidptosis with traditional chemoradiotherapy also warrant further investigation. Additionally, it is crucial to explore whether inducing disulfidptosis alters the immune microenvironment, as combining disulfidptosis inducers with immunotherapy (e.g., PD‐1/PD‐L1 antibodies) could enhance tumor eradication. Moreover, combining disulfidptosis inducers with targeted drug delivery systems to selectively kill cancer cells while minimizing off‐target effects and toxicity is an exciting future prospect. Besides, disulfidptosis is not only applicable to cancer therapy but also shows potential in the treatment of neurological disorders^19^, ^20^ and bone metabolism abnormalities.^21^ Exploring the application of disulfidptosis in nontumor fields is also crucial.

In conclusion, inducing disulfidptosis holds tremendous potential for future clinical treatments. Collaborative efforts across disciplines are essential to accelerate the research on disulfidptosis mechanisms and advance its clinical translation.

## AUTHOR CONTRIBUTIONS

Tai Mi led the conceptualization of the review theme, played a primary role in drafting the manuscript, and coordinated the literature review process. Xiangpan Kong and Meiling Chen participated in the drawing and modification of pictures and collected and sorted out relevant literature. Peng Guo guided the content of the article and participated in the revision of the article. Dawei He Supervised the entire project, ensured the accuracy and completeness of the work, and participated in the critical review and revision of manuscripts. All authors read and approved the final manuscript.

## CONFLICT OF INTEREST STATEMENT

The authors declare no conflict of interest.

## ETHICS STATEMENT

Not applicable.

## Data Availability

Data sharing does not apply to this study.
